# Genomic resources for population analyses of an invasive insect pest *Oryctes rhinoceros*

**DOI:** 10.1038/s41597-023-02109-y

**Published:** 2023-04-11

**Authors:** Igor Filipović

**Affiliations:** 1 The University of Queensland, School of Biological Sciences, St. Lucia, Australia; 2 QIMR Berghofer Medical Research Institute, Herston, Australia

**Keywords:** Data integration, Data processing

## Abstract

Over the last few years, various types of NGS data have been accumulating for the coconut rhinoceros beetle (CRB, *Oryctes rhinoceros*), reflecting the growing interest in curtailing this invasive pest of palm trees. Whilst reference-free analyses of RNA-seq and RAD-seq datasets have been done for different CRB collections, recent availability of the CRB’s genome assembly provides an opportunity to collate diverse data and create a reference-based population dataset. Here, I release such a dataset containing 6,725,935 SNPs and genotypes called across 393 individual samples from 16 populations, using the previously published raw sequences generated in 9 different experiments (RAD-Seq, RNA-Seq, WGS). I also provide reference-based datasets for the CRB’s mitochondrial variants and for variants of its viral biocontrol agent Oryctes rhinoceros nudivirus. SNP data provide high resolution for determining the geographic origin of invasive CRB. With these genomic resources, new data can be analysed without re-processing the published samples and then integrated to expand the reference datasets.

## Background and Summary

The coconut rhinoceros beetle (CRB), *Oryctes rhinoceros*, has been responsible for major yield losses of coconut and oil palms across its native and invasive range^1^. Invasive CRB populations across the South Pacific islands were suppressed for decades with a largely non-chemical toolbox that included the use of an entomopathogenic virus, Oryctes rhinoceros nudivirus (OrNV)^2^. In recent years, however, incursions of *O. rhinoceros* were recorded in territories that were previously free of this pest, from Guam, Hawai’i and Solomon Islands^3,4^ to Vanuatu and New Caledonia^5^. Different hypotheses have been proposed to explain the resurgence of *O. rhinoceros*, one being beetle’s increased tolerance to the OrNV’s pathogenicity, associated with a particular mitochondrial lineage (known as the “CRB-G” biotype) that is common in the newly-invaded regions^6^. However, new data show that OrNV infections are common in the CRB-G lineage^7,8^, and some evidence supports the hypothesis of an emergent OrNV strain that is less-virulent^8^. Understanding the mechanisms behind the breakdown of a formerly-successful biocontrol strategy is critical for re-establishing effective suppression of this pest and for preventing its future invasions.

Population genomics and phylogenetics provide a good framework for testing different hypotheses around CRB invasions, including interactions with its biocontrol agent (OrNV), through the analyses of genetic patterns of both biological systems in space and time. The power to resolve alternative hypotheses within this framework depends on the ability to grow the reference dataset for CRB populations and OrNV isolates, with spatial and temporal collections spanning native and invasive range.

Over the last few years, next-generation sequencing (NGS) experiments have been done for different CBR collections, accumulating data from the reduced-representation sequencing (Restriction site-Associated DNA sequencing, RAD-Seq; Genotyping-by-Sequencing, GBS)^7,9^, as well as RNA sequencing (RNA-Seq)^10–13^ and Whole Genome Sequencing (WGS)^14–16^. Until recently, however, absence of a reference genome assembly for CRB’s nuclear and mitochondrial genomes have prevented the direct comparison and collation of different datasets. Whilst population genomic analyses of CRB’s RAD-Seq data have been done in a reference-free manner (i.e. using a *de novo* approach^17^), data from different experiments that use different restriction enzymes cannot be directly compared without being aligned to the reference genome. Data comparison between different technologies, like RNA-Seq and RAD-Seq, is also impractical without reference-aligned data.

Recently-released high-quality genome assemblies for CRB’s nuclear^18^ and mitochondrial^19^ genomes provide an opportunity to collate all NGS data generated for this pest thus far and to create a reference-based population dataset. I fully re-processed raw sequencing data from 393 CRB samples from 16 countries, that included data from RAD-Seq, WGS and RNA-Seq experiments deposited within 9 BioProjects in the NCBI’s Sequence Read Archive (Metadata Table^20^). I generated a dataset^20^ with 6,725,935 Single Nucleotide Polymorphisms (SNPs) and 636,134 indels, and showed that the called genotypes provide high resolution for determining population genetic structure and history. I also provide SNP and indel dataset^20^ for the CRB mitochondrial genome variation, and for variation found in the isolates of its biocontrol agent, OrNV. With these genomic resources^20^, researchers will be able to fully utilize the existing population data, and have a reference point to further increase the sampling resolution in space and time.

## Methods

### NGS data collation - CRB datasets

I retrieved all NGS data generated from the CRB samples that were deposited to the National Center for Biotechnology Information (NCBI) BioProject database and were publicly available in January 2022. There were nine BioProjects that included raw Illumina sequences from:

- four Whole Genome Sequencing (WGS) datasets: one described as a WGS data from the mitochondrial genome (PRJNA735922^21^), two as the OrNV samples (PRJNA682856^22^, PRJNA413966^23^), and one as a whole-organism sample (PRJNA724335^24^)
- two Restriction-site Associated DNA sequencing (RAD-Seq) datasets: one generated with restriction enzymes NlaIII and MluCI (PRJNA433414^25^), one generated with PstI and MspI (PRJNA648153^26^)
- three RNA sequencing (RNA-Seq) datasets: PRJNA486419^27^, PRJNA547367^28^, PRJNA639990^29^.

**Fig. 1:**
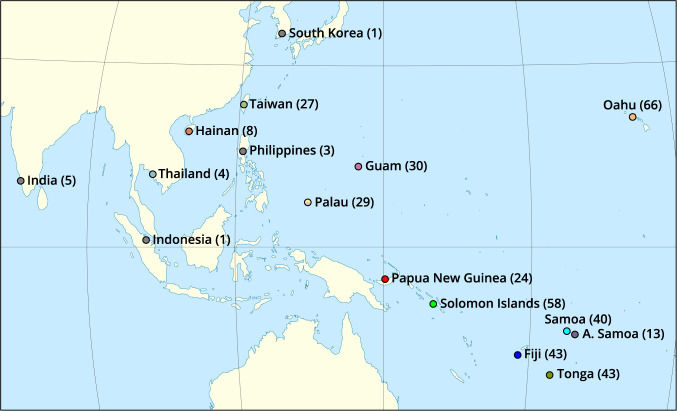
Sampling locations and CRB sample sizes (in brackets). The map is sourced and build upon the World Map Winkel Tripel projection centered on the 150 degrees East, by Eric Gaba under Attribution-ShareAlike 3.0 Unported License (CC BY-SA 3.0) (https://commons.wikimedia.org/w/index.php?curid = 16501581).

In total, there were 393 samples collected from 16 countries/territories (Fig. 1): American Samoa, Fiji, Guam, Hainan (China), India, Indonesia, Oahu (Hawai’i), Palau, Papua New Guinea, Philippines, Samoa, Solomon Islands, South Korea, Taiwan, Thailand, and Tonga. The details on the BioProjects and the number of raw sequences per each CRB sample are listed in the Metadata Table^20^.

### Data QC and processing

Trimmomatic^30^ v.0.39 was used to remove Illumina adapters from the sequencing reads and to discard low quality sequences from further analyses, using the following parameters:


SE -phred33 inputN.fastq trimmedN.fastq ILLUMINACLIP:Illumina.fa:2:30:10 MINLEN:35


The number of sequences per sample following the processing step is found in the Metadata Table^20^. On average, 99.97% of raw sequences were retained after this processing step, indicating the high-quality of CRB sequences deposited in NCBI.

### Sequence mapping

The processed sequences were first mapped to the OrNV genomic sequence (NCBI: MN623374.1^31,32^), the remaining sequences were then mapped to the CRB mitochondrial genome sequence (NCBI: MT457815.1^19,33^), and then to the CRB nuclear genome sequences (NCBI: GCA_020654165.1^18,34^).

DNA sequences (WGS and RAD-Seq data) were mapped with Bowtie 2^35^ v2.4.4, using the following parameters:

(for paired-end data)


--phred33 --end-to-end --very-sensitive -q --minins 35 --maxins 1000 --no-discordant --no-unal -x assembly-index -1 trimmedN_1.fastq -2 trimmedN_2.fastq -S outputN.sam


(for single-end data)


--phred33 --end-to-end --very-sensitive -q --no-unal -x assembly-index -U trimmedN.fastq -S outputN.sam


RNA sequences were mapped with HISAT2^36^ v2.1.0, using the following parameters:


-q -k 5 --min-intronlen 20 --max-intronlen 20000 --no-mixed --no-discordant --no-unal -x assembly-index -1 trimmedN_1.fastq -2 trimmedN_2.fastq -S outputN.sam


The mapping process for each of the three genomes (CRB nuclear, mitochondrial and OrNV) produced three sets of.sam files. All.sam files from one set (nuclear/mitochondrial/OrNV) were converted to the sorted and indexed.bam files with SAMTools^37^ v1.13. using the following parameters:


view -b -T assembly.fasta -o outputN.bam outputN.samsort -o sorted.outputN.bam outputN.bamindex sorted.outputN.bam


All individual files for each dataset were then merged into one indexed merged.bam file using the SAMtools v1.13 commands:


merge -c -p -o merged.sorted.output.bam sorted.output1.bam sorted.output2.bam sorted.output3.bam … sorted.outputN.bamindex -b merged.sorted.output.bam


### Realignment of mapped sequences

In order to improve the detection of variants in the downstream process, I used ABRA2^38^ v2.23 to realign the mapped sequences that have been merged from all samples in each of the three sets (nuclear/mitochondrial/OrNV), using the following parameters:


--ref assembly.fasta --in merged.sorted.output.bam --out realigned.bam --mapq 0 --nosort --no-edge-ci


The final.bam file containing realigned sequences was indexed with SAMtools^39^ v1.13


index -b realigned.bam


### Variant and genotype calling

Bcftools^39^ v1.13 was used for variant (SNP and indel) and genotype calling, and for producing the variant call format (VCF) file for each dataset (CRB nuclear, CRB mitochondrial, and OrNV) using the following commands:


mpileup --output-type u --fasta-ref assembly.fasta --max-depth 393000 --min-BQ 13 --config illumina --output assembly-pileup.bcf realigned.bamcall --multiallelic-caller -mv -Oz -o assembly.vcf.gz assembly-pileup.bcf


## Data Records

The genomic resources produced in this study are the publicly-accessible VCF files:

1. GCA_020654165.1_S4-7k-2v3_genomic.vcf.gz^20,40^
  with ~6.7 M SNP and ~600 K indel positions in the CRB nuclear genome assembly with genotypes scored across 393 CRB individuals.
2. MT457815.1_S4_mitochondrion.vcf.gz^20,40^
  with 562 SNP and 36 indel positions in the CRB mitochondrial genome
3. MN623374.1_S4_OrNV.vcf.gz^20,40^
  with 6458 SNP and 356 indel positions in the OrNV genome.
4. Metadata_table.xlsx^20^

containing the information for all 393 CRB samples used in this study, including: the sequencing strategy, sample name (re-coded for this study), the number of raw Illumina sequences, the number of sequences passing the QC step, the number of sequences mapped with Bowtie 2 on each of the three assemblies as well as the number of unmapped sequences (for RAD-Seq and WGS sequencing data), the number of sequences mapped with HISAT2 on each of the three assemblies as well as, the number of unmapped sequences (for RNA-Seq data), sample sequences origin composition in percentages (OrNV, *O. Rhinoceros* and undetermined), percent of coverage per assembly, original sample ID, NCBI Run, NCBI experiment, NCBI SRA study, NCBI BioProject and NCBI BioProject description.

All data records are available in figshare^20^ and the variant data produced in this study is also deposited in the European Variation Archive^41^ (EVA) at EMBL-EBI under accession number PRJEB59266^40^.

## Technical Validation

### SNPs in the CRB and OrNV genomes have the expected Transition and Transversion ratio (Ts/Tv)

A commonly used metric for assessing the overall quality of SNP calling is the Transition and Transversion ratio (Ts/Tv)^42^, For human genome data, the Ts/Tv ratio is between 2 and 3, depending on whether the analysed SNPs are inside or outside exons^42^. Using the program Vcftools^43^, I obtained the Ts/Tv ratio of 2.08 for SNPs in the CRB nuclear genome dataset (GCA_020654165.1_S4-7k-2v3_genomic.vcf^20,40^), the ratio of 2.99 for the CRB mitogenome dataset (MT457815.1_S4_mitochondrion.vcf^20,40^), and the ratio of 2.62 for the OrNV dataset (MN623374.1_S4_OrNV.vcf^20,40^), all of which indicate high overall quality of SNPs reported in these datasets.

### Reference-based phylogeny is concordant with geography and known CRB history

**Fig. 2:**
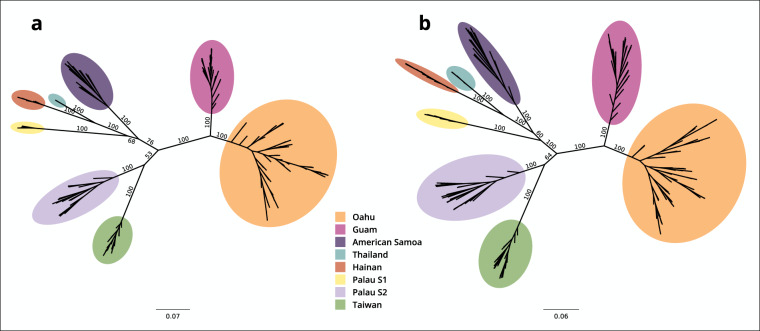
Maximum likelihood (ML) phylogeny for (**a**) the reference-free CRB dataset from Reil *et al*.^9^ and (**b**) the reference-based dataset with the samples from the same study^9^.

To validate the quality of the reference-based SNP dataset for testing different hypotheses around CRB invasion history, I compared the maximum likelihood (ML) phylogeny generated with this dataset^20^ to the previously-published phylogeny generated with the reference-free data^44^. Specifically, I reproduced the reference-free phylogeny by Reil *et al*.^9^ reported in their Fig. 4B,by following the reported steps for raw sequence data processing, de novo SNP and genotype calling, and variant and sample filtering^44^. The final dataset contained genotype calls for 151 CRB individuals across 7,037 variable sites, with site missingness of ≤ 0.5 (STA151 dataset in Reil *et al*.^9^). The vcf2phylip.py^45^ script was used to transform the data from the VCF file to the PHYLIP multiple sequence alignment format (with heterozygous genotypes represented as IUPAC ambiguities), and the ML phylogeny was produced with the program RAxML-NG^46^. I successfully reproduced the originally-reported ML tree that has geographic samples clearly separated as highly supported branches (Oahu, Guam, American Samoa, Hainan, Thailand, Taiwan, with Palau splitting into two lineages, Fig. 2A).

The reference-based dataset (GCA_020654165.1_S4-7k-2v3_genomic.vcf^20,40^) was filtered to contain all 172 individuals from the Reil *et al*. study^9^, and SNP data only (indels were removed), where at least 166 individuals had to have a genotype at each variable position (site missingness ≤ 0.05). This filtering produced the final dataset with genotype calls at 15,699 sites in 166 individuals (6 individuals were excluded due to having > 0.97 missing data). As with the reference-free dataset, genotype calls were transformed into the PHYLIP file using the vcf2phylip.py^45^ script and the ML phylogeny was produced with RAxML-NG^46^.

The resulting ML phylogeny (Fig. 2b) confirmed that the reference-based SNP data generated in this study can be reliably used to infer CRB population history. The reference-based tree had the same topology as the previously published tree (Fig. 2b vs 2a) that is concordant with the known invasion pathway of this pest^5^. Using the reference-aligned reads, genotypes can be more reliably called in individuals with low sequencing depth when compared to *de novo* (reference-free) genotype calling. Consequently, more individuals and loci can be included in the analysis despite having lower sequencing depth when the reference genome is available. In the example above, reference-free (*de novo*) analysis included 155 CRB individuals with ~7 K SNP, while the reference-based analysis contained 166 individuals with~15 K SNPs, Moreover, having a reference genome enables identification and removal of contaminant sequences that could cause erroneous SNP calling. In the Reil *et al*.^9^ dataset, 6 CRB individuals had to be excluded from the analysis, despite having good numbers of sequencing reads, because more than 80% of their sequences were identified as exogenous i.e. could not be aligned to the CRB nuclear or mitochondrial genomes, indicating severe sample contamination and/or degradation.

### Reference-based data are concordant between different RAD-Seq experiments

The reference-based dataset (GCA_020654165.1_S4-7k-2v3_genomic.vcf^20,40^) was filtered to contain individuals from two different RAD-Seq experiments: (1) Reil *et al*.^9^, where the library was prepared using two frequently cutting restriction enzymes (MluCI, NlaIII), and from (2) Etebari *et al*.^7^, where the library was prepared using one frequently cutting restriction enzyme (MspI) and one less frequently cutting enzyme (PstI). The final dataset contained 3,802 SNP sites with a minor allele frequency of 0.05 and missingness < 0.3 across 356 CRB individuals from 12 locations. 14 individuals were removed from the analysis due to not having a genotype at > 80% of the selected SNP sites. Genotype calls were transformed into the PHYLIP file using the vcf2phylip.py^45^ script and the ML phylogeny was produced with RAxML-NG^46^.

**Fig. 3:**
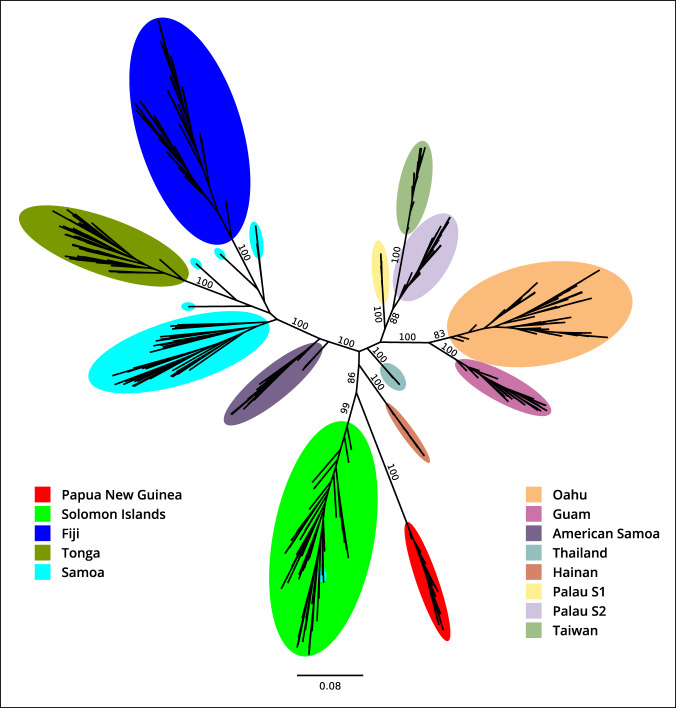
Maximum likelihood (ML) phylogeny for the collated reference-based dataset with the CRB samples from different RAD-Seq experiments (Reil *et al*.^9^, Etebari *et al*.^7^).

The resulting ML tree (Fig. 3) shows that the reference-based data collated from different NGS experiments produce a robust inference of the relationship between CRB populations. For example, population samples from Samoa and American Samoa, although sequenced in different experiments, form the closely-related branches on one of the major lineages that also includes Fiji and Tonga (Fig. 3). This indicates that the batch effect of the technology/experiments is minimal (if present at all) in comparison to the biological signal reflecting geography and population history, further validating the quality of the reference-based data.

### Reference-based data from different technologies (RAD-Seq and RNA-Seq) give concordant signals of population structure

To confirm that the reference-based SNP data obtained from DNA sequencing is concordant with the data from RNA sequencing, I analysed the CRB samples collected from three geographic locations (Fiji, Papua New Guinea, Solomon Islands). The reference-based dataset (GCA_020654165.1_S4-7k-2v3_genomic.vcf^20,40^) was filtered to contain 8 individuals previously analysed in a RNA-Seq study by Etebari *et al*.^12^, and 99 individuals previously analysed in a RAD-Seq experiment by Etebari *et al*.^7^. The final dataset contained 1,828 SNP sites with a minor allele frequency of 0.05 and missingness of 0.05. Discriminant Analysis of Principal Components (DAPC)^47^ was done with the R package adegenet^48^ v.1.42.

**Fig. 4:**
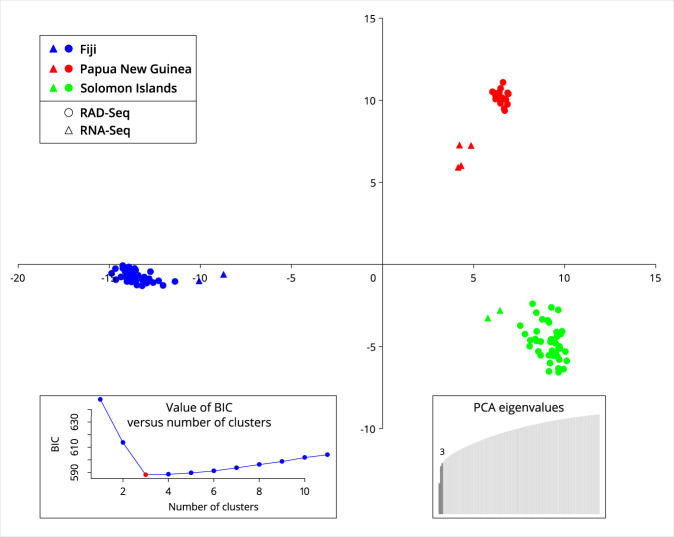
DAPC for the collated reference-based CRB dataset from the RNA-Seq and RAD-Seq experiments by Etebari *et al*.^7,13^.

DAPC correctly inferred three genetic clusters (“best” K = 3 using the BIC criterion, Fig. 4) without describing the population groups *a priori*. These genetic clusters are clearly separated along the first two discriminant axes even when a minimal number of principal components (3) was retained in order to avoid data over-fitting (PCA eigenvalues, Fig. 4). Assignment of individuals reflected their geographic origin and not the sequencing technology, thus validating the quality of the reference-based genotype calls for the samples that are sequenced with different approaches. It is worth noting that individuals from the RNA-Seq experiment were positioned closer to the centre of the vector space than individuals from the RAD-Seq experiment (Fig. 4). This is because individuals from this RNA-seq experiment had more missing data at the chosen SNP positions, and these missing data were replaced with the mean allele frequencies during the analysis of principal components^47,48^ which resulted in their positioning closer to the mean value (centre) of the vector space. Nevertheless, a strong biological signal from the scored genotypes in these individuals led to their unambiguous grouping by population origin, confirming that the reference-called variants are of high quality for inferring population genetic structure.

## Usage Notes

VCF files^20,40^ generated in this study allow researchers to compare new CRB and OrNV data with the existing data without re-processing the published samples. To expand the reference datasets for population analyses, researchers should generate the reference-based VCF files following the steps presented in the Methods section and merge their VCF files with the VCF files from this study using e.g. BCFtools^39^. Researchers can also use VCF files^20,40^ provided here to realign their mapped sequences, which should improve indel detection in particular, using e.g. ABRA2^38^. To best utilise the maximum number of existing samples and variants for population genomic analyses under stronger budgetary constraints, researchers should prepare their NGS libraries using the RAD-Seq approach with the frequently cutting restriction enzymes NlaIII and MluCI and fragment size selection as in Reil *et al*.^9^. Where permitted by higher research budgets, new data generated *via* Whole Genome Sequencing are optimal for further integration, as this NGS approach provides complete information on both CRB genomes (nuclear and mitochondrial) and on the genome of the biocontrol agent OrNV.

## Data Availability

For ease of access and reproducibility, the Methods and Technical Validation sections contain the command parameters and referencing for all versions of third-party software and scripts used in this study.
